# Two years later: Is the SARS-CoV-2 pandemic still having an impact on emergency surgery? An international cross-sectional survey among *WSES* members

**DOI:** 10.1186/s13017-022-00424-0

**Published:** 2022-06-16

**Authors:** Martin Reichert, Massimo Sartelli, Markus A. Weigand, Matthias Hecker, Philip U. Oppelt, Julia Noll, Ingolf H. Askevold, Juliane Liese, Winfried Padberg, Federico Coccolini, Fausto Catena, Andreas Hecker, The WSES COVID-19 emergency surgery survey collaboration group, Adam Peckham-Cooper, Adrian Camacho-Ortiz, Aikaterini T. Mastoraki, Aitor Landaluce-Olavarria, Ajay Kumar Pal, Akira Kuriyama, Alain Chichom-Mefire, Alberto Porcu, Aleix Martínez-Pérez, Aleksandar R. Karamarkovic, Aleksei V. Osipov, Alessandro Coppola, Alessandro Cucchetti, Alessandro Spolini, Alessio Giordano, Alexander Reinisch-Liese, Alfie J. Kavalakat, Alin Vasilescu, Amin Alamin, Amit Gupta, Ana Maria Dascalu, Ana-Maria Musina, Anargyros Bakopoulos, Andee Dzulkarnaen Zakaria, Andras Vereczkei, Andrea Balla, Andrea Bottari, Andreas Baumann, Andreas Fette, Andrey Litvin, Aniella Katharina Reichert, Anna Guariniello, Anna Paspala, Anne-Sophie Schneck, Antonio Brillantino, Antonio Pesce, Arda Isik, Ari Kalevi Leppäniemi, Aristeidis Papadopoulos, Aristotelis Kechagias, Ashraf Yehya Abdalla Mohamed, Ashrarur Rahman Mitul, Athanasios Marinis, Athanasios Syllaios, Baris Mantoglu, Belinda De Simone, Benjamin Stefan Weiss, Bernd Pösentrup, Biagio Picardi, Biagio Zampogna, Boris Eugeniev Sakakushev, Boyko Chavdarov Atanasov, Bruno Nardo, Bulent Calik, Camilla Cremonini, Carlos A. Ordoñez, Charalampos Seretis, Chiara Cascone, Christos Chouliaras, Cino Bendinelli, Claudia Lopes, Claudio Guerci, Clemens Weber, Constantinos Nastos, Cristian Mesina, Damiano Caputo, Damien Massalou, Davide Cavaliere, Deborah A. McNamara, Demetrios Demetriades, Desirè Pantalone, Diego Coletta, Diego Sasia, Diego Visconti, Dieter G. Weber, Diletta Corallino, Dimitrios Chatzipetris, Dimitrios K. Manatakis, Dimitrios Ntourakis, Dimitrios Papaconstantinou, Dimitrios Schizas, Dimosthenis Chrysikos, Dmitry Mikhailovich Adamovich, Doaa Elkafrawy, Dragos Seban, Edgar Fernando Hernandez García, Edoardo Baldini, Edoardo Picetti, Edward C. T. H. Tan, Efstratia Baili, Eftychios Lostoridis, Elena Adelina Toma, Elif Colak, Elisabetta Cerutti, Elmin Steyn, Elmuiz A. Hsabo, Emmanouil Ioannis Kapetanakis, Emmanouil Kaouras, Emmanuel Schneck, Emrah Akin, Emre Gonullu, Enes çelik, Enrico Cicuttin, Enrico Pinotti, Erik Johnsson, Ernest E. Moore, Ervis Agastra, Evgeni Nikolaev Dimitrov, Ewen A. Griffiths, Fabrizio D’Acapito, Federica Saraceno, Felipe Alconchel, Felix Alexander Zeppernick, Fernando Machado Rodríguez, Fikri Abu-Zidan, Francesca Pecchini, Francesco Favi, Francesco Ferrara, Francesco Fleres, Francesco Pata, Francesco Pietro Maria Roscio, Francesk Mulita, Frank J. M. F. Dor, Fredrik Linder, Gabriel Dimofte, Gabriel Rodrigues, Gabriela Nita, Gabriele Sganga, Gennaro Martines, Gennaro Mazzarella, Gennaro Perrone, George Velmahos, Georgios D. Lianos, Gia Tomadze, Gian Luca Baiocchi, Giancarlo D’Ambrosio, Gianluca Pellino, Gianmaria Casoni Pattacini, Giorgio Giraudo, Giorgio Lisi, Giovanni Domenico Tebala, Giovanni Pirozzolo, Giulia Montori, Giulio Argenio, Giuseppe Brisinda, Giuseppe Currò, Giuseppe Giuliani, Giuseppe Palomba, Giuseppe Roscitano, Gökhan Avşar, Goran Augustin, Guglielmo Clarizia, Gustavo M. Machain Vega, Gustavo P. Fraga, Harsheet Sethi, Hazim Abdulnassir Eltyeb, Helmut A. Segovia Lohse, Herald René Segovia Lohse, Hüseyin Bayhan, Hytham K. S. Hamid, Igor A. Kryvoruchko, Immacolata Iannone, Imtiaz Wani, Ioannis I. Lazaridis, Ioannis Katsaros, Ioannis Nikolopoulos, Ionut Negoi, Isabella Reccia, Isidoro Di Carlo, Iyiade Olatunde Olaoye, Jacek Czepiel, Jae Il Kim, Jeremy Meyer, Jesus Manuel Saenz Terrazas, Joel Noutakdie Tochie, Joseph M. Galante, Justin Davies, Kapil Sugand, Kebebe Bekele Gonfa, Kemal Rasa, Kenneth Y. Y. Kok, Konstantinos G. Apostolou, Konstantinos Lasithiotakis, Konstantinos Tsekouras, Kumar Angamuthu, Lali Akhmeteli, Larysa Sydorchuk, Laura Fortuna, Leandro Siragusa, Leonardo Pagani, Leonardo Solaini, Lisa A. Miller, Lovenish Bains, Luca Ansaloni, Luca Ferrario, Luigi Bonavina, Luigi Conti, Luis Antonio Buonomo, Luis Tallon-Aguilar, Lukas Tomczyk, Lukas Werner Widmer, Maciej Walędziak, Mahir Gachabayov, Maloni M. Bulanauca, Manu L. N. G. Malbrain, Marc Maegele, Marco Catarci, Marco Ceresoli, Maria Chiara Ranucci, Maria Ioanna Antonopoulou, Maria Papadoliopoulou, Maria Rosaria Valenti, Maria Sotiropoulou, Mario D’Oria, Mario Serradilla Martín, Markus Hirschburger, Massimiliano Veroux, Massimo Fantoni, Matteo Nardi, Matti Tolonen, Mauro Montuori, Mauro Podda, Maximilian Scheiterle, Maximos Frountzas, Mehmet Sarıkaya, Mehmet Yildirim, Michael Bender, Michail Vailas, Michel Teuben, Michela Campanelli, Michele Ammendola, Michele Malerba, Michele Pisano, Mihaela Pertea, Mihail Slavchev, Mika Ukkonen, Miklosh Bala, Mircea Chirica, Mirko Barone, Mohamed Maher Shaat, Mohammed Jibreel Suliman Mohammed, Mona Awad Akasha Abuelgasim, Monika Gureh, Mouaqit Ouadii, Mujdat Balkan, Mumin Mohamed, Musluh Hakseven, Natalia Velenciuc, Nicola Cillara, Nicola de’Angelis, Nicolò Tamini, Nikolaos J. Zavras, Nikolaos Machairas, Nikolaos Michalopoulos, Nikolaos N. Koliakos, Nikolaos Pararas, Noel E. Donlon, Noushif Medappil, Offir Ben-Ishay, Olmi Stefano, Omar Islam, Ömer Tammo, Orestis Ioannidis, Oscar Aparicio, Oussama Baraket, Pankaj Kumar, Pasquale Cianci, Per Örtenwall, Petar Angelov Uchikov, Philip de Reuver, Philip F. Stahel, Philip S. Barie, Micaela Piccoli, Piotr Major, Pradeep H. Navsaria, Prakash Kumar Sasmal, Raul Coimbra, Razrim Rahim, Recayi Çapoğlu, Renol M. Koshy, Ricardo Alessandro Teixeira Gonsaga, Riccardo Pertile, Rifat Ramadan Mussa Mohamed, Rıza Deryol, Robert G. Sawyer, Roberta Angelico, Roberta Ragozzino, Roberto Bini, Roberto Cammarata, Rosa Scaramuzzo, Rossella Gioco, Ruslan Sydorchuk, Salma Ahmed, Salomone Di Saverio, Sameh Hany Emile, Samir Delibegovic, Sanjay Marwah, Savvas Symeonidis, Scott G. Thomas, Sebahattin Demir, Selmy S. Awad, Semra Demirli Atici, Serge Chooklin, Serhat Meric, Sevcan Sarıkaya, Sharfuddin Chowdhury, Shaza Faycal Mirghani, Sherry M. Wren, Simone Gargarella, Simone Rossi Del Monte, Sofia Esposito, Sofia Xenaki, Soliman Fayez Ghedan Mohamed, Solomon Gurmu Beka, Sorinel Lunca, Spiros G. Delis, Spyridon Dritsas, Stefan Morarasu, Stefano Magnone, Stefano Rossi, Stefanos Bitsianis, Stylianos Kykalos, Suman Baral, Sumita A. Jain, Syed Muhammad Ali, Tadeja Pintar, Tania Triantafyllou, Tarik Delko, Teresa Perra, Theodoros A. Sidiropoulos, Thomas M. Scalea, Tim Oliver Vilz, Timothy Craig Hardcastle, Tongporn Wannatoop, Torsten Herzog, Tushar Subhadarshan Mishra, Ugo Boggi, Valentin Calu, Valentina Tomajer, Vanni Agnoletti, Varut Lohsiriwat, Victor Kong, Virginia Durán Muñoz-Cruzado, Vishal G. Shelat, Vladimir Khokha, Wagih Mommtaz Ghannam, Walter L. Biffl, Wietse Zuidema, Yasin Kara, Yoshiro Kobe, Zaza Demetrashvili, Ziad A. Memish, Zoilo Madrazo, Zsolt J. Balogh, Zulfu Bayhan

**Affiliations:** 1 Department of General, Visceral, Thoracic, Transplant and Pediatric Surgery, University Hospital of Giessen, Rudolf-Buchheim-Strasse 7, 35392 Giessen, Germany; 2 Department of Surgery, Macerata Hospital, Macerata, Italy; 3 Department of Anesthesiology, Heidelberg University Hospital, Heidelberg, Germany; 4 Department of Pulmonary and Critical Care Medicine, University Hospital of Giessen and Marburg Lung Center (UGMLC), University Hospital of Giessen, Giessen, Germany; 5 Department of General, Emergency and Trauma Surgery, Pisa University Hospital, Pisa, Italy; 6 Department of Emergency Surgery, Parma Maggiore Hospital, Parma, Italy

**Keywords:** COVID-19, SARS-CoV-2, Pandemic, Emergency surgery, Emergency, Appendicitis, WSES, Time to intervention, Capacity, Quarantine

## Abstract

**Background:**

The SARS-CoV-2 pandemic is still ongoing and a major challenge for health care services worldwide. In the first *WSES COVID-19 emergency surgery survey*, a strong negative impact on emergency surgery (ES) had been described already early in the pandemic situation. However, the knowledge is limited about current effects of the pandemic on patient flow through emergency rooms, daily routine and decision making in ES as well as their changes over time during the last two pandemic years. This second *WSES COVID-19 emergency surgery survey* investigates the impact of the SARS-CoV-2 pandemic on ES during the course of the pandemic.

**Methods:**

A web survey had been distributed to medical specialists in ES during a four-week period from January 2022, investigating the impact of the pandemic on patients and septic diseases both requiring ES, structural problems due to the pandemic and time-to-intervention in ES routine.

**Results:**

367 collaborators from 59 countries responded to the survey. The majority indicated that the pandemic still significantly impacts on treatment and outcome of surgical emergency patients (83.1% and 78.5%, respectively). As reasons, the collaborators reported decreased case load in ES (44.7%), but patients presenting with more prolonged and severe diseases, especially concerning perforated appendicitis (62.1%) and diverticulitis (57.5%). Otherwise, approximately 50% of the participants still observe a delay in time-to-intervention in ES compared with the situation before the pandemic. Relevant causes leading to enlarged time-to-intervention in ES during the pandemic are persistent problems with in-hospital logistics, lacks in medical staff as well as operating room and intensive care capacities during the pandemic. This leads not only to the need for triage or transferring of ES patients to other hospitals, reported by 64.0% and 48.8% of the collaborators, respectively, but also to paradigm shifts in treatment modalities to non-operative approaches reported by 67.3% of the participants, especially in uncomplicated appendicitis, cholecystitis and multiple-recurrent diverticulitis.

**Conclusions:**

The SARS-CoV-2 pandemic still significantly impacts on care and outcome of patients in ES. Well-known problems with in-hospital logistics are not sufficiently resolved by now; however, medical staff shortages and reduced capacities have been dramatically aggravated over last two pandemic years.

## Background

Since the World Health Organization has declared SARS-CoV-2 as a worldwide pandemic in early 2020, it dominates daily life and influences political decisions as well as daily therapeutic practices in various medical disciplines. Global or local lockdown policies and recommendations regarding postponement of elective therapies during the last two pandemic years aim at providing necessary health care capacities especially during wave-like outbreaks of different variants of SARS-CoV-2. Therefore, the discussion behind postponements of elective surgical therapies is primarily to hold up bed, intensive care as well as operating room capacities available during the pandemic and secondarily to preserve patients and medical staff from nosocomial infection with SARS-CoV-2 [1–4]. However, postponement of elective surgical therapies might not be detrimental in the short-term follow-up upon diagnosis, but delayed treatments might have some harmful effects beyond that short range in the longer-term outcome [2]. This it is quite clear for surgical oncology, because delays in cancer-specific therapy might result in severely impaired oncologic outcome [2, 5–7].Even patient outcome is impaired through postponements of elective surgical therapy of other non-malignant diseases during the pandemic [8]. The situation is dramatically different in emergency patients. Postponement of urgent therapies is not allowed in the emergency setting and can rapidly result in detrimental outcome [9, 10]. However, local hospital management for maintaining an adequate emergency surgical service during wave-like SARS-CoV-2 outbreaks with high incidences of severely-ill COVID-19 patients and limited intensive care capacities is challenging. Policies, health care providers and hospital management have to balance the necessity of strong SARS-CoV-2 measures on the one hand and the maintenance of surgical emergency services on the other hand, which both compete with critical hospital resources including normal ward, intensive care and operating room capacities as well as personnel required for an adequate therapy and additional expenses for quarantine measures.

Since the rapid spread of the SARS-CoV-2 around the world in late 2019, a high amount of research had been published regarding the pandemic, the characteristics of the virus and treatment modalities of COVID-19, which are urgently needed [4]. However, the literature reveals some evidence about dramatic negative effects of the pandemic on various medical fields and health care services, e.g., in oncology, cardiovascular diseases, etc., as well as on the outcome of respective patients [5, 11–14]. By now, this list is endless and the SARS-CoV-2 pandemic has obviously a negative impact on emergency surgery worldwide: Although case load decreases, patients present with more severe and prolonged surgical emergencies, and thus, treatment is more complex and surgery is more complicated during the pandemic [3, 15, 16].

As a consequence, these pandemic-driven factors uncertainly influence not only the entire spectrum of elective patient care, but also have a significant effect on daily routine patient care in emergency rooms and particularly in emergency surgery. However, the knowledge is limited about the impact of the pandemic on patient flow through emergency rooms, clinical daily routine and clinical decision making in emergency surgery as well as their changes over time during the last two years of the pandemic. In our first *WSES COVID-19 emergency surgery survey* from June 2020, we have described a strong impact of the pandemic on emergency surgery services worldwide due to potential harmful delays in time-to-diagnosis (TTD) and time-to-intervention (TTI), and the need of triage of emergency surgical patients [3]. Data analysis revealed that structural problems like in-hospital logistics were predominantly responsible for negative effects of SARS-CoV-2 on emergency surgical patient care [3]. This new second *WSES COVID-19 emergency surgery survey* investigates the impact of the SARS-CoV-2 pandemic on patients and their diseases requiring emergency surgery, on TTD and TTI in emergency departments as well as relevant causes for a delayed surgical therapy during the course of the pandemic. Two years after the initial survey structural rearrangement, new in-hospital standard operating procedures and integration of SARS-CoV-2 into the everyday clinicians’ work should have led to an improvement of emergency care. If the initial problems persist, this must lead to a radical and immediate improvement of surgical emergency care worldwide.

## Methods

An online survey was designed by a core group of investigators of the study group. Google Forms (Google LLC, Mountain View, California, USA) was used as the platform for the survey.

The survey was designed, the survey study was conducted, and the results were analyzed and reported following the CHERRIES statement [17].

The survey consists of multiple-choice and single-choice items as well as open-answer questions. In the style of the first *WSES COVID-19 emergency surgery survey* study from June 2020, published in December 2020 [3], the items of the present questionnaire are organized in five sections: (1) recording the characteristics of collaborators and their affiliated hospitals, (2) evaluating the experiences of the study group with emergency surgery in COVID-19-infected patients, (3) investigating the ongoing impact of SARS-CoV-2 pandemic on patients requiring emergency surgery as well as (4) ongoing structural problems caused by the pandemic and leading to substantial barriers across emergency surgical pathways and quality loss in emergency patient treatment. Furthermore, (5) individual experiences with septic diseases potentially requiring intensive care, interventional emergency therapy and/or emergency surgery as well as (6) potential paradigm shifts from a standard operative therapy before the pandemic situation to a non-operative (conservative) treatment approach during the pandemic and (7) preventive, quarantine and SARS-CoV-2 testing strategies are part of the survey.

After the survey was approved by the *World Society of Emergency Surgery* (WSES) project steering committee, this cross-sectional survey study was distributed on January 17, 2022, to the global mailing list of WSES members and beyond, as addressees were invited to distribute the survey to their colleagues. The survey was closed after four weeks on February 11, 2022.

After closing the survey, the results were checked for duplicates. Results are presented descriptively within the present manuscript.

Results of items, that were comparable with our previous *WSES COVID-19 emergency surgery survey* study from June 2020 [3], were conducted to statistical analyses, which allowed us to investigate changes in responses over time during the pandemic situation. Statistical analyses were performed using GraphPad Prism (Version 9 for Windows, GraphPad Software, San Diego, CA, USA, www.graphpad.com). For descriptive statistics, categorical data were analyzed using Fishers exact test or Pearson’s *X*^2^ test. *p* values ≤ 0.05 were considered to indicate statistical significance. Because of the exploratory character of the study, no adjustments of p values were performed.

Data are given as *n* collaborators and/or percentages of the collaborators.

## Results

### Characteristics of participants and their affiliated hospitals

**Fig. 1:**
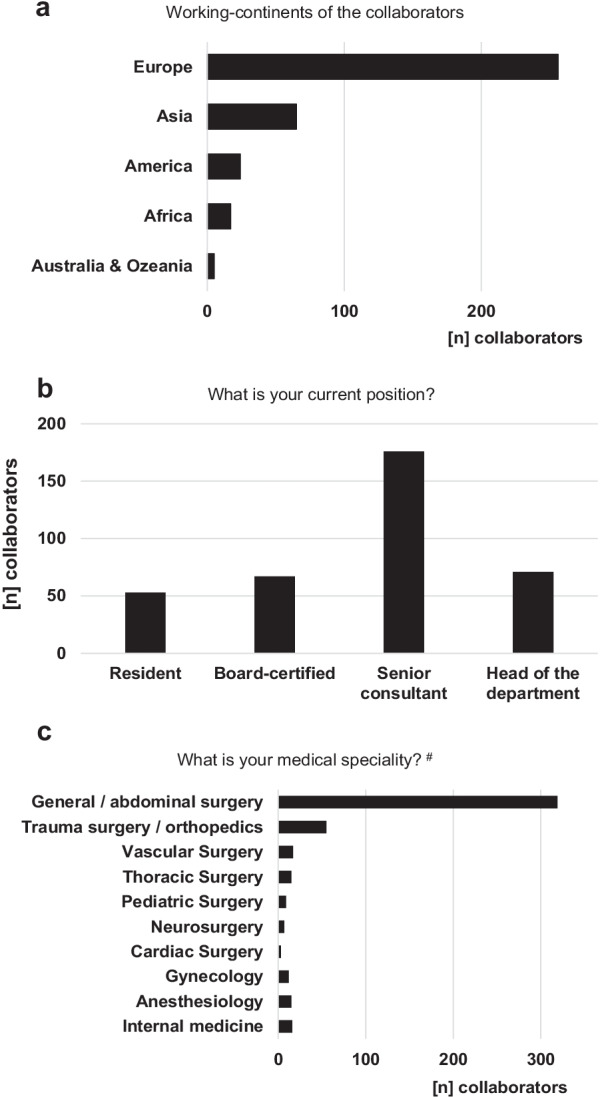
Characteristics of the collaborators. Working continents of the *WSES COVID-19 emergency surgery survey collaboration group* (**a**). Experience and position (**b**) as well as medical speciality (**c**) of the collaborators. #multiple answers possible

**Fig. 2:**
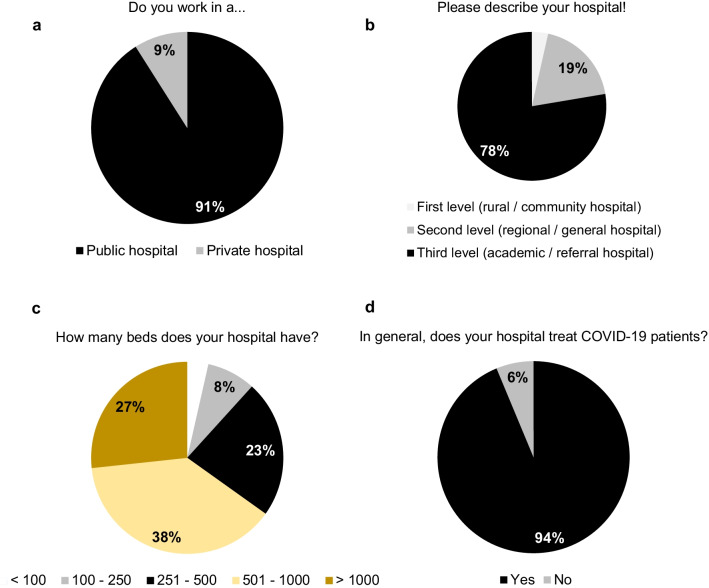
Hospital characteristics of the collaboration group. Volume and academic affiliation (**a**–**c**) of the hospitals as well as reported treatment capacities of COVID-19 patients (**d**)

367 health care specialists in emergency medicine and emergency surgical patient care from 59 countries around the world, i.e., 5 continents, completed the survey study (Fig. 1). Most participants work in a larger public, third-level (academic/referral) hospital with more than 500 beds (Fig. 2) which includes a dedicated emergency department (96.2% of the cases) and more precisely a dedicated acute care surgery or emergency surgery unit at the own hospital (71.9% of the cases). With 86.9% and 15%, most collaborators came from general/abdominal surgery and trauma surgery/orthopedics, respectively, but representatives of other medical disciplines, which are involved in emergency surgical patient care, were also represented in the study cohort including internal medicine, anesthesiology, vascular surgery, thoracic surgery and gynecology (all < 5%). Therefore, most of the collaborators are well-experienced personnel with being senior consultants and heads of the departments in 48.0% and 19.3%, respectively, or at least board-certified staff in 18.3% (Fig. 1).

**Fig. 3:**
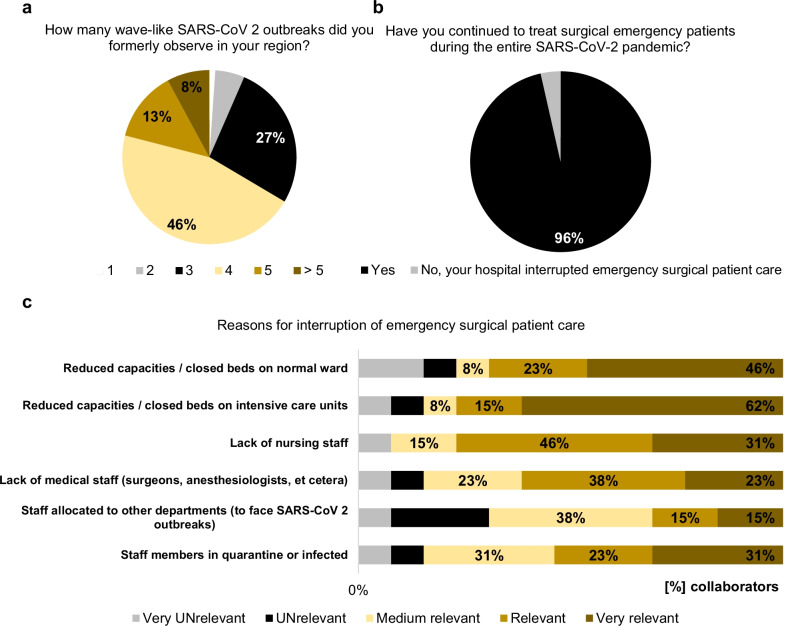
SARS-CoV-2 outbreaks and emergency surgery. Interruption of surgical emergency patient care in response to SARS-CoV-2 outbreaks (**a**, **b**) as well as reasons for interruption of surgical emergency patient care during the pandemic (**c**)

93.5% of the participants stated that they formally experienced three or more SARS-CoV-2 outbreaks in their region since the beginning of the pandemic situation in early 2020 (Fig. 3) and almost all collaborators (94%) stated that their region currently (from October 2021 to February 2022) suffers from a wave-like SARS-CoV-2 outbreak. In general, COVID-19 patients are treated in hospitals of 93.7% of the collaborators (Fig. 2) and almost all of the collaborators (88%) had performed emergency surgery in patients, acutely infected with SARS-CoV-2, which represents a significant increase over time compared with the situation in June 2020 (versus 62.2%, *p* < 0.0001 [3]). Interestingly, in hospitals of 13 participants (3.5%) emergency surgical patient care was interrupted due to the pandemic. Relevant or very relevant reasons for a discontinuous emergency surgery service among these participants were: reduced capacities on normal ward (69.2%) and intensive care units (76.9%), lack of nursing (76.9%) and medical staff (61.5%) as well as staff members being in quarantine or infected (53.8%, Fig. 3).

### Ongoing impact of the pandemic on capacities and quality indicators of emergency surgery

**Fig. 4:**
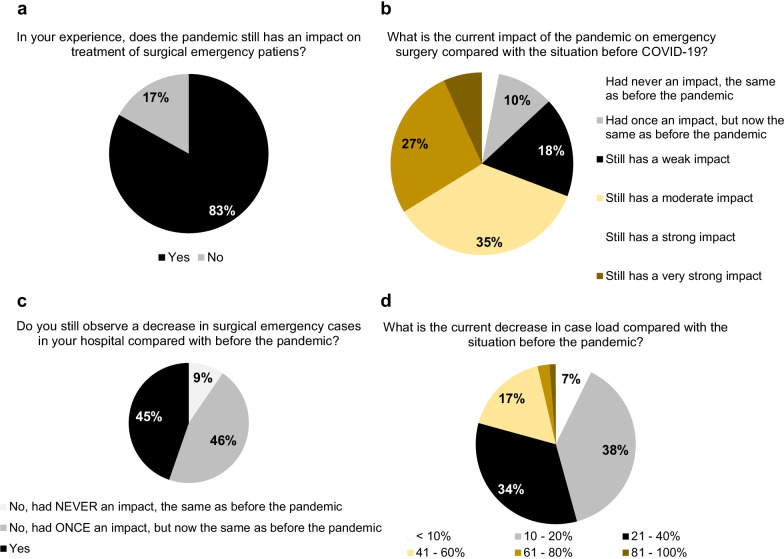
The impact of the pandemic situation on emergency surgery. The estimated “global” impact of the pandemic on emergency surgery (**a**, **b**) and the effect of the pandemic situation on emergency surgical case load (**c**, **d**)

In the previous *WSES COVID-19 emergency surgery survey* in June 2020, we reported that the SARS-CoV-2 pandemic negatively influences the treatment of surgical emergency patients strongly [3]. Therefore, 83.1% of the collaborators indicated now that the pandemic still has an impact on treatment of surgical emergency patients and estimated this impact as being strong or very strong in 33.8% of the cases (Fig. 4). Interestingly, 18 of the 23 participants, from hospitals, which are not involved in care of SARS-CoV-2 positive patients, also observe these negative effects. However, the negative impact of the SARS-CoV-2 pandemic on emergency surgical services due to the pandemic was fortunately reduced over time compared with answers from the first *WSES COVID-19 emergency surgery survey* in June 2020, where almost 65.5% of the participants estimated it as being strong or very strong (*p* < 0.0001 [3]). Overall 44.7% of the participants still observe a decrease in emergency surgical cases in their hospitals compared with the situation before the pandemic (versus 86.2% of the collaborators from the previous *WSES COVID-19 emergency surgery survey* in June 2020, *p* < 0.0001 [3]). However, 74.1% of the participants reported this effect being  mostly obvious during local lockdown policies.

**Fig. 5:**
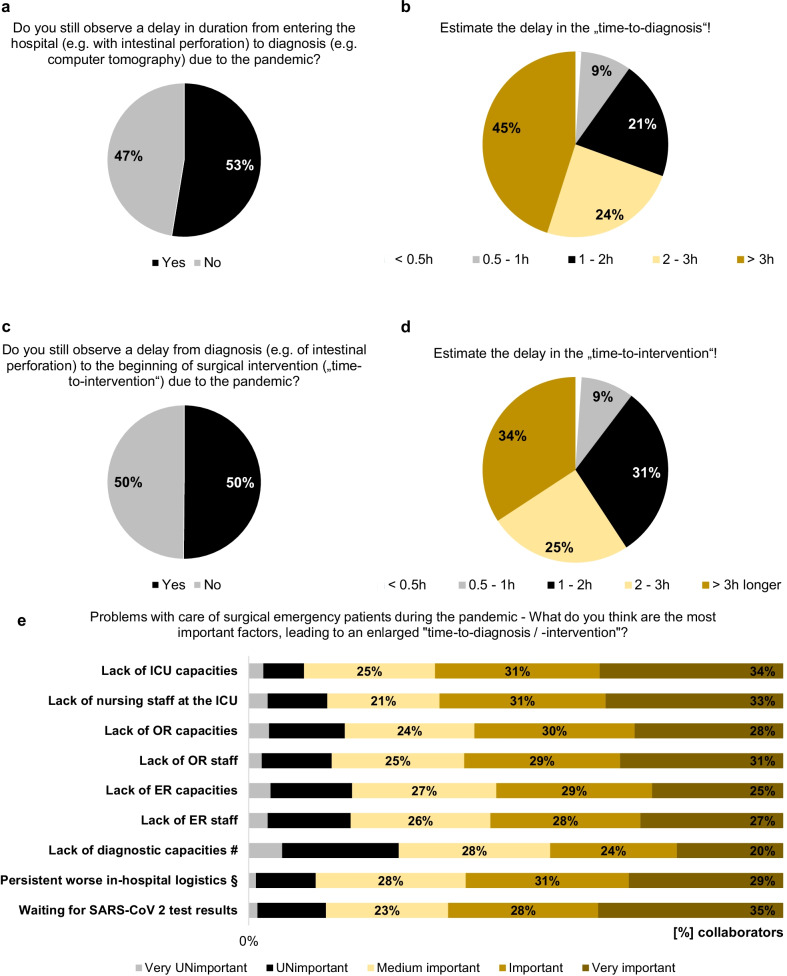
Delays in surgical emergency patient care. Do the collaborators still observe delays in time-to-diagnosis (**a**, **b**) and time-to-intervention (**c**, **d**) in emergency surgery and the reasons behind these observations (**e**). ^#^Lack of diagnostic capacities (e.g., computed tomography, endoscopy, etc.). ^§^Persistent worse in-hospital logistics (e.g., transport of patients, closed normal wards, etc.)

**Fig. 6:**
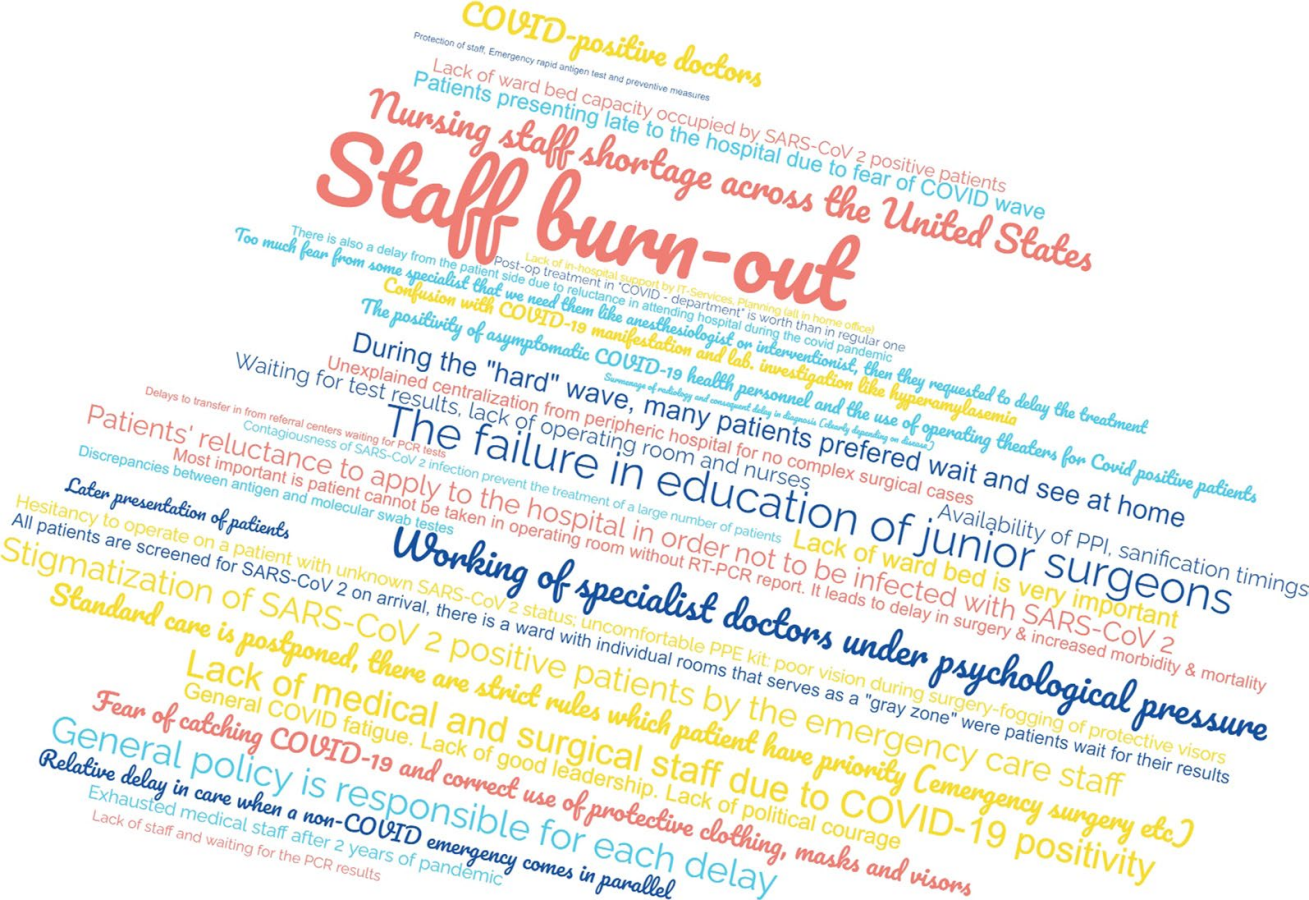
Factors leading to problems with surgical emergency patient care during the pandemic. Free-text answers in a word cloud from the collaborators collecting relevant factors, which lead to problems with surgical emergency patient care during the pandemic

Important quality indicators for emergency patient care are TTD and TTI. In the current survey, 52.6% and 50.1% of the participants still observe a delay in TTD and consecutively in TTI, respectively, due to the pandemic, which was comparable with the situation in June 2020 (*p* = 0.0734 and *p* = 0.2331 [3]). Even no obvious differences were seen in the estimated delays in TTD (*p* = 0.3763 [3]) and TTI (*p* = 0.0582 [3]) over time during the SARS-CoV-2 pandemic. To evaluate relevant factors being critical in the therapy of surgical emergency patients and why the SARS-CoV-2 pandemic might have an ongoing impact on emergency room and emergency surgical pathways, the collaborators of the study were asked for the most important factors that still lead to an enlarged TTD and TTI as well as problems with capacities concerning emergency surgery during the pandemic situation. The answers are summarized in Figs. 5e and 6. Interestingly, we did not observe improvements in in-hospital logistics, e.g., for transport of emergency patients or separated pathways for SARS-CoV-2-infected patients, reported over time from the previous *WSES COVID-19 emergency surgery survey* in June 2020 until now (*p* = 1 [3]), which is until now one of the most important problems for delays in TTD and TTI. Vice versa, significantly more collaborators currently report a dramatic lack in medical staff, especially in the operating room, leading to both unavailable operating room and intensive care unit capacities (all of them *p* < 0.0001 in comparison with the responses from the first *WSES COVID-19 emergency surgery survey* in June 2020 [3]), indicating that staff shortages and reduced capacities, relevant for emergency surgery, are dramatically aggravated during the course of the SARS-CoV-2 pandemic over the last two years. These problems with limited local capacities continuously result in a relevant need of triage or transferring surgical emergency patients to other hospitals for further therapy, reported by 64% and 48.8% of the collaborators, respectively. Figure 6 reports free-text answers from the collaborators collecting further factors, which lead to problems with surgical emergency patient care during the pandemic.

### Ongoing impact of SARS-CoV-2 pandemic on diseases requiring critical care, emergency therapy, emergency surgery

**Fig. 7:**
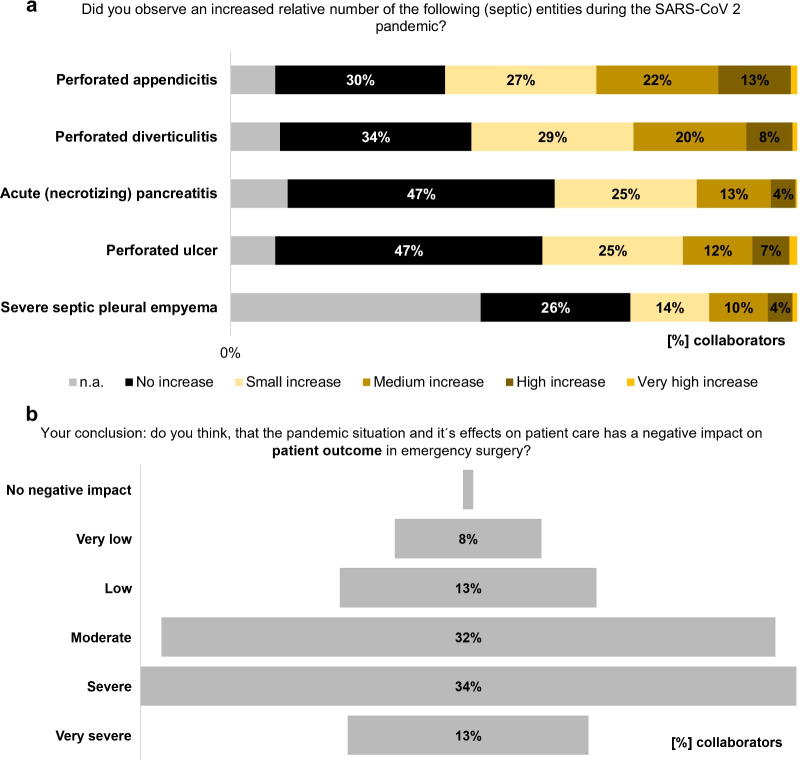
The effect of the pandemic on patient outcome. The impact of the SARS-CoV-2 pandemic on diseases requiring emergency surgery (**a**) and on outcome of surgical emergency patients (**b**)

**Fig. 8:**
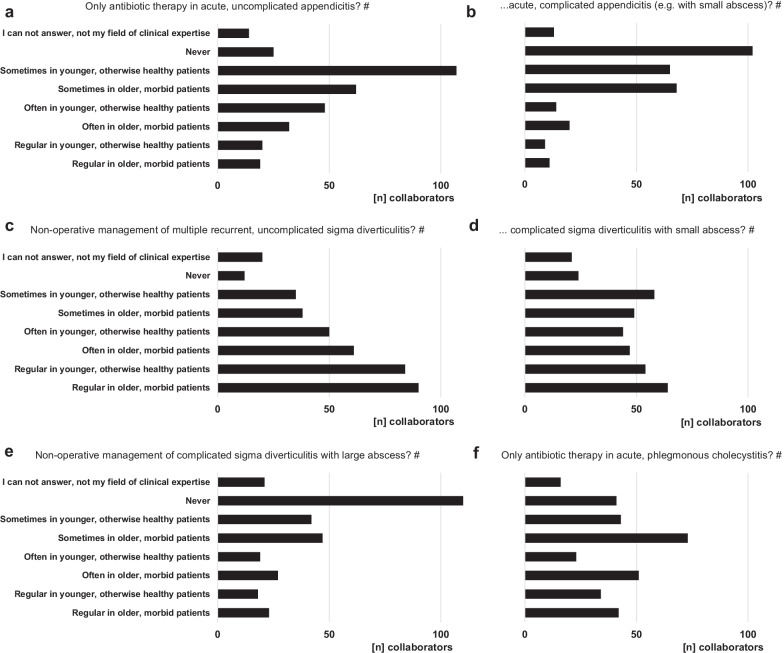
Paradigm shift in treatment modalities. Shift of treatment modalities due to the pandemic from a frequently intended surgical therapy before to a non-operative approach during the pandemic situation in patients with appendicitis (**a**, **b**), diverticulitis (**c**–**e**) and cholecystitis (**f**). ^#^multiple answers possible

The collaborators of the current *WSES COVID-19 emergency surgery survey* observed an ongoing medium, high or very high increase in relative numbers of severe (septic) abdomino-thoracic diseases, especially of perforated appendicitis and diverticulitis, during the pandemic in comparison with the situation before (Fig. 7a); however, this was comparable with the estimation of the participants from June 2020 (perforated appendicitis: *p* = 0.5586 and perforated diverticulitis: *p* = 1328 [3]). Contrastingly, two-thirds of the collaborators (67.3%) reported their experience that the pandemic situation (with reduced capacities, higher perioperative risk, etc.) led to a paradigm shift in the treatment of more uncomplicated infectious diseases to a non-operative approach. These findings are underlined by the reports upon the questions, which non-operative treatment options the participants recommend to their patients during the ongoing pandemic situation. This was mostly obvious for acute, uncomplicated infectious diseases including appendicitis and diverticulitis, but also more complicated diseases were considered to more conservative treatment modalities during the pandemic by a remarkable proportion of participants. Therefore, patient characteristics play an important role in these treatment considerations (Fig. 8).

In a conclusion, most of the collaborators think that the pandemic situation and its effects on emergency surgical patient care have a moderate (31.9%), severe (34.1%) or very severe (12.5%) negative impact on emergency surgical patient outcome. Only 13.4%, 7.6% and 0.5% of the participants think that this negative impact on patient outcome in the surgical emergency setting due to pandemic-driven changes is low, very low or zero, respectively (Fig. 7b). Interestingly, all participants, from hospitals, which are not involved in care of SARS-CoV-2 positive patients (*n* = 23), also estimated a moderate (*n* = 12), severe (*n* = 9) or very severe (*n* = 2) negative impact on the outcome of emergency surgical patients.

### Preventive and testing strategies

**Fig. 9:**
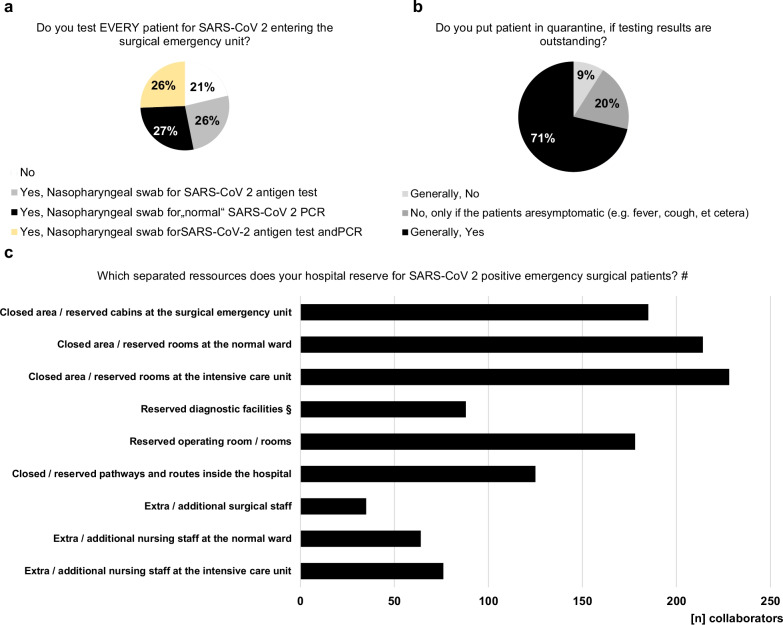
Testing and quarantine strategies. Local testing (**a**) and quarantine strategies (**b**) varying strongly. (**c**) Which separated pathways are useful and which resources are provided by hospital management to separate SARS-CoV-2 positive from uninfected patients. ^#^multiple answers possible. ^§^Reserved diagnostic facilities (e.g., computed tomography scanner, ultrasonography, etc.)

In the hospitals of 78.7% of the collaborators, every patient entering the surgical emergency unit undergoes SARS-CoV-2 testing and 71.4% put patients in quarantine generally, if testing results are outstanding (Fig. 9). Therefore, “rapid” SARS-CoV-2-PCR is available at the surgical emergency unit of 76.3% of the participants for urgent results within one hour. 89.4% of the collaborators reported that their hospitals provide separated pathways for SARS-CoV-2 positive patients to minimize contact with uninfected patients. The most commonly used measures are closed areas and reserved rooms at the emergency unit, normal wards and intensive care units as well as reserved operating theatres for SARS-CoV-2 positive patients.

## Discussion

The SARS-CoV-2 pandemic has still devastating effects on surgical emergency services worldwide. Our initial *WSES COVID-19 emergency surgery survey* from June 2020 revealed in the very early pandemic phase that there is a strong impact of the pandemic situation on surgical emergency patient care including potential harmful delays in TTD and TTI. This led either to a delayed therapy or the urge of triage of surgical emergency patients in a relevant proportion worldwide [3]. At that early time point, structural problems with in-hospital logistics were mainly responsible for these problems in patient care, whereas medical staff shortages previously did not play a major role contributing to these challenges in emergency surgery [3]. The current cross-sectional survey reflects the situation with emergency surgical patient care presently, approximately 2 years after. By now, the pandemic is still ongoing and various regions of the world suffered from numerous wave-like outbreaks of diverse variants of SARS-CoV-2. However, beyond several heterogeneous strategies to maintain global health care during the pandemic including social lockdown policies, adjusted treatment recommendations and in-hospital quarantine measures, the negative effect of the pandemic situation on surgical emergency patient care and on patient outcome is still significant. These alarming findings are underlined by persistent delays in TTD and TTI for emergency surgical patients during the last two years of the pandemic, which had been reported by approximately 50% of the collaborators. Therefore, it is well known that rapid initiation of diagnostics and therapy especially for patients with septic diseases in emergency surgery and trauma patients is mandatory for good treatment quality [9, 10, 18–20]. Furthermore, the collaborators were asked for relevant reasons causing the significant impact of the pandemic situation on emergency surgery: Persistent problems with in-hospital logistics are still major factors making an adequate surgical emergency treatment impossible. Obviously, these problems could not be resolved during the two-year lasting pandemic situation. Based on the results of the first *WSES COVID-19 emergency surgery survey* in 2020, we declared critical impairments in the diagnostic and therapeutic work-up of surgical emergency patients as a worldwide issue and postulated that improvements in care of surgical emergency patients like tailored solutions for in-hospital logistics are urgently necessary [3, 21]. Furthermore, we then hypothesized that proactive provision of resources to enhance medical staff, operating room capacities, intensive care capacities from policies, medical societies and local health care providers are mandatory for future patient care during the pandemic [3, 21]. In contrast, the situation in surgical emergency units worldwide is aggravated by dramatic shortages in medical staff, capacities and resources, which consecutively deteriorates the problematic situation with surgical emergency patients during the pandemic. Both the well-known logistic problems paired with the “new” lack of (human) resources summit in an enlarged TTD and TTI in many cases and finally to impaired outcome of our patients by now. Nevertheless, one has to keep in mind that some of these culprits, especially those logistic ones, might be modifiable easily, which might lead to significant improvements in in-hospital emergency patient care.

Another important aspect of the pandemic situation are changes in patient flow into the surgical emergency departments and changes in the disease quality requiring emergency surgery over the last two years. Overall a decreasing case load of emergency surgical patients is reported since the beginning of the pandemic in both *WSES COVID-19 emergency surgery surveys* [3]. But, an increase in more severe and prolonged diseases, including perforated appendicitis and diverticulitis, requiring more complex surgical or multimodal treatment strategies has been observed. Although it is a subjective estimation from the participants of the present survey, this finding is not entirely novel and had also been described by other authors from larger scaled cross-sectional studies involving patient data in the literature before. Although overall patient numbers are decreasing, especially patients with bowel diseases including appendicitis and diverticulitis present in the emergency rooms with more severe, prolonged and even perforated diseases [3, 8, 15, 16]. Besides that, Tebala et al. showed in their international retrospective cohort audit correlations of hospital admissions with general patient condition and physical health [22]. These changes in patient flow into surgical emergency departments as well as the observation of higher proportion of patients with more severe and prolonged diseases might be an expression of ongoing changes in medical evaluation and decision-making processes and a kind of pandemic-driven fear reactions of patients and referring physicians against SARS-CoV-2 infection [23]. Other issues, indicating changes in medical evaluation and particularly in decision-making processes, are paradigm shifts in the treatment modalities of frequent infectious diseases, which classically require (general) emergency surgery. Particularly in acute appendicitis, multiple recurrent and symptomatic non-complicative diverticulitis and cholecystitis a great proportion of participants would refer on a non-operative management, which—vice versa—was not regularly the case before the pandemic [24]. Additionally, some participants of the survey stated to apply non-operative therapeutic strategies for other severe septic diseases, including primary drainage therapy for perforated peptic ulcer in higher aged and polymorbid patients and prolonged drainage therapy to avoid—if possible—decortication for pleural empyema. If these circumstances will change treatment perceptions and therapeutic standards beyond the special situation during the pandemic remains to be seen in the future, however, emergency surgeons have to be aware that discussions are ongoing regarding more conservative treatment strategies in selected cases especially with appendicitis and diverticulitis.

In contrast, patients with traumatic emergencies frequently do not tolerate treatment postponements or conservative therapy attempts. The estimation of the collaborators, that the pandemic results in impaired outcomes of emergency surgical patients, underlines the essential necessity to maintain adequate and continuous patient care within the emergency setting including sufficient in-hospital and personnel capacities as well as appropriate in-hospital logistics. The latter includes fast SARS-CoV-2 testing (79% of the participants are testing every patient entering the surgical emergency room) and consequent quarantine measures with closed and reserved areas for patients with doubted or confirmed SARS-CoV-2 infection as highly important strategies on the one hand to prevent patients from misdiagnosis and delayed initiation of adequate therapy. On the other hand, these measures are mandatory to prevent patients and medical staff from nosocomial SARS-CoV-2 infection during high-incidence periods, which would worsen shortages in emergency surgical patient care, exhaustion and psychological pressure of personnel dramatically.

## Conclusions

In conclusion, the current study demonstrates that the SARS-CoV-2 pandemic still has a significant and global impact on care and outcome of surgical emergency patients. Well-known problems with in-hospital logistics are not sufficiently resolved by now; however, medical staff shortages and reduced capacities have been dramatically aggravated during the pandemic situation over the last two years. Global strategies to maintain adequate emergency surgical patient care are urgently needed from policies and hospital management [21]. These measures are not only mandatory during the ongoing SARS-CoV-2 pandemic and future outbreaks of virus variants but also for prospective disaster management protocols.

## Data Availability

The datasets used and/or analyzed during the current study are available from the corresponding author on reasonable request.

## Abbreviations

CHERRIES: The Checklist for Reporting Results of Internet E-Surveys
COVID-19: Corona virus disease 2019
PCR: Polymerase chain reaction
SARS-CoV-2: Severe acute respiratory syndrome corona virus type 2
TTD: Time-to-diagnosis
TTI: Time-to-intervention
WSES: World Society of Emergency Surgery
