# Correction to: Two years later: Is the SARS-CoV-2 pandemic still having an impact on emergency surgery? An international cross-sectional survey among *WSES* members

**DOI:** 10.1186/s13017-022-00442-y

**Published:** 2022-07-08

**Authors:** Martin Reichert, Massimo Sartelli, Markus A. Weigand, Matthias Hecker, Philip U. Oppelt, Julia Noll, Ingolf H. Askevold, Juliane Liese, Winfried Padberg, Federico Coccolini, Fausto Catena, Andreas Hecker, The WSES COVID-19 emergency surgery survey collaboration group, Adam Peckham-Cooper, Adrian Camacho-Ortiz, Aikaterini T. Mastoraki, Aitor Landaluce-Olavarria, Ajay Kumar Pal, Akira Kuriyama, Alain Chichom-Mefire, Alberto Porcu, Aleix Martínez-Pérez, Aleksandar R. Karamarkovic, Aleksei V. Osipov, Alessandro Coppola, Alessandro Cucchetti, Alessandro Spolini, Alessio Giordano, Alexander Reinisch-Liese, Alfie J. Kavalakat, Alin Vasilescu, Amin Alamin, Amit Gupta, Ana Maria Dascalu, Ana-Maria Musina, Anargyros Bakopoulos, Andee Dzulkarnaen Zakaria, Andras Vereczkei, Andrea Balla, Andrea Bottari, Andreas Baumann, Andreas Fette, Andrey Litvin, Aniella Katharina Reichert, Anna Guariniello, Anna Paspala, Anne-Sophie Schneck, Antonio Brillantino, Antonio Pesce, Arda Isik, Ari Kalevi Leppäniemi, Aristeidis Papadopoulos, Aristotelis Kechagias, Ashraf Yehya Abdalla Mohamed, Ashrarur Rahman Mitul, Athanasios Marinis, Athanasios Syllaios, Baris Mantoglu, Belinda De Simone, Benjamin Stefan Weiss, Bernd Pösentrup, Biagio Picardi, Biagio Zampogna, Boris Eugeniev Sakakushev, Boyko Chavdarov Atanasov, Bruno Nardo, Bulent Calik, Camilla Cremonini, Carlos A. Ordoñez, Charalampos Seretis, Chiara Cascone, Christos Chouliaras, Cino Bendinelli, Claudia Lopes, Claudio Guerci, Clemens Weber, Constantinos Nastos, Cristian Mesina, Damiano Caputo, Damien Massalou, Davide Cavaliere, Deborah A. McNamara, Demetrios Demetriades, Desirè Pantalone, Diego Coletta, Diego Sasia, Diego Visconti, Dieter G. Weber, Diletta Corallino, Dimitrios Chatzipetris, Dimitrios K. Manatakis, Dimitrios Ntourakis, Dimitrios Papaconstantinou, Dimitrios Schizas, Dimosthenis Chrysikos, Dmitry Mikhailovich Adamovich, Doaa Elkafrawy, Dragos Serban, Edgar Fernando Hernandez García, Edoardo Baldini, Edoardo Picetti, Edward C. T. H. Tan, Efstratia Baili, Eftychios Lostoridis, Elena Adelina Toma, Elif Colak, Elisabetta Cerutti, Elmin Steyn, Elmuiz A. Hsabo, Emmanouil Ioannis Kapetanakis, Emmanouil Kaouras, Emmanuel Schneck, Emrah Akin, Emre Gonullu, Enes çelik, Enrico Cicuttin, Enrico Pinotti, Erik Johnsson, Ernest E. Moore, Ervis Agastra, Evgeni Nikolaev Dimitrov, Ewen A. Griffiths, Fabrizio D’Acapito, Federica Saraceno, Felipe Alconchel, Felix Alexander Zeppernick, Fernando Machado Rodríguez, Fikri Abu-Zidan, Francesca Pecchini, Francesco Favi, Francesco Ferrara, Francesco Fleres, Francesco Pata, Francesco Pietro Maria Roscio, Francesk Mulita, Frank J. M. F. Dor, Fredrik Linder, Gabriel Dimofte, Gabriel Rodrigues, Gabriela Nita, Gabriele Sganga, Gennaro Martines, Gennaro Mazzarella, Gennaro Perrone, George Velmahos, Georgios D. Lianos, Gia Tomadze, Gian Luca Baiocchi, Giancarlo D’Ambrosio, Gianluca Pellino, Gianmaria Casoni Pattacini, Giorgio Giraudo, Giorgio Lisi, Giovanni Domenico Tebala, Giovanni Pirozzolo, Giulia Montori, Giulio Argenio, Giuseppe Brisinda, Giuseppe Currò, Giuseppe Giuliani, Giuseppe Palomba, Giuseppe Roscitano, Gökhan Avşar, Goran Augustin, Guglielmo Clarizia, Gustavo M. Machain Vega, Gustavo P. Fraga, Harsheet Sethi, Hazim Abdulnassir Eltyeb, Helmut A. Segovia Lohse, Herald René Segovia Lohse, Hüseyin Bayhan, Hytham K. S. Hamid, Igor A. Kryvoruchko, Immacolata Iannone, Imtiaz Wani, Ioannis I. Lazaridis, Ioannis Katsaros, Ioannis Nikolopoulos, Ionut Negoi, Isabella Reccia, Isidoro Di Carlo, Iyiade Olatunde Olaoye, Jacek Czepiel, Jae Il Kim, Jeremy Meyer, Jesus Manuel Saenz Terrazas, Joel Noutakdie Tochie, Joseph M. Galante, Justin Davies, Kapil Sugand, Kebebe Bekele Gonfa, Kemal Rasa, Kenneth Y. Y. Kok, Konstantinos G. Apostolou, Konstantinos Lasithiotakis, Konstantinos Tsekouras, Kumar Angamuthu, Lali Akhmeteli, Larysa Sydorchuk, Laura Fortuna, Leandro Siragusa, Leonardo Pagani, Leonardo Solaini, Lisa A. Miller, Lovenish Bains, Luca Ansaloni, Luca Ferrario, Luigi Bonavina, Luigi Conti, Luis Antonio Buonomo, Luis Tallon-Aguilar, Lukas Tomczyk, Lukas Werner Widmer, Maciej Walędziak, Mahir Gachabayov, Maloni M. Bulanauca, Manu L. N. G. Malbrain, Marc Maegele, Marco Catarci, Marco Ceresoli, Maria Chiara Ranucci, Maria Ioanna Antonopoulou, Maria Papadoliopoulou, Maria Rosaria Valenti, Maria Sotiropoulou, Mario D’Oria, Mario Serradilla Martín, Markus Hirschburger, Massimiliano Veroux, Massimo Fantoni, Matteo Nardi, Matti Tolonen, Mauro Montuori, Mauro Podda, Maximilian Scheiterle, Maximos Frountzas, Mehmet Sarıkaya, Mehmet Yildirim, Michael Bender, Michail Vailas, Michel Teuben, Michela Campanelli, Michele Ammendola, Michele Malerba, Michele Pisano, Mihaela Pertea, Mihail Slavchev, Mika Ukkonen, Miklosh Bala, Mircea Chirica, Mirko Barone, Mohamed Maher Shaat, Mohammed Jibreel Suliman Mohammed, Mona Awad Akasha Abuelgasim, Monika Gureh, Mouaqit Ouadii, Mujdat Balkan, Mumin Mohamed, Musluh Hakseven, Natalia Velenciuc, Nicola Cillara, Nicola de’Angelis, Nicolò Tamini, Nikolaos J. Zavras, Nikolaos Machairas, Nikolaos Michalopoulos, Nikolaos N. Koliakos, Nikolaos Pararas, Noel E. Donlon, Noushif Medappil, Offir Ben-Ishay, Olmi Stefano, Omar Islam, Ömer Tammo, Orestis Ioannidis, Oscar Aparicio, Oussama Baraket, Pankaj Kumar, Pasquale Cianci, Per Örtenwall, Petar Angelov Uchikov, Philip de Reuver, Philip F. Stahel, Philip S. Barie, Micaela Piccoli, Piotr Major, Pradeep H. Navsaria, Prakash Kumar Sasmal, Raul Coimbra, Razrim Rahim, Recayi Çapoğlu, Renol M. Koshy, Ricardo Alessandro Teixeira Gonsaga, Riccardo Pertile, Rifat Ramadan Mussa Mohamed, Rıza Deryol, Robert G. Sawyer, Roberta Angelico, Roberta Ragozzino, Roberto Bini, Roberto Cammarata, Rosa Scaramuzzo, Rossella Gioco, Ruslan Sydorchuk, Salma Ahmed, Salomone Di Saverio, Sameh Hany Emile, Samir Delibegovic, Sanjay Marwah, Savvas Symeonidis, Scott G. Thomas, Sebahattin Demir, Selmy S. Awad, Semra Demirli Atici, Serge Chooklin, Serhat Meric, Sevcan Sarıkaya, Sharfuddin Chowdhury, Shaza Faycal Mirghani, Sherry M. Wren, Simone Gargarella, Simone Rossi Del Monte, Sofia Esposito, Sofia Xenaki, Soliman Fayez Ghedan Mohamed, Solomon Gurmu Beka, Sorinel Lunca, Spiros G. Delis, Spyridon Dritsas, Stefan Morarasu, Stefano Magnone, Stefano Rossi, Stefanos Bitsianis, Stylianos Kykalos, Suman Baral, Sumita A. Jain, Syed Muhammad Ali, Tadeja Pintar, Tania Triantafyllou, Tarik Delko, Teresa Perra, Theodoros A. Sidiropoulos, Thomas M. Scalea, Tim Oliver Vilz, Timothy Craig Hardcastle, Tongporn Wannatoop, Torsten Herzog, Tushar Subhadarshan Mishra, Ugo Boggi, Valentin Calu, Valentina Tomajer, Vanni Agnoletti, Varut Lohsiriwat, Victor Kong, Virginia Durán Muñoz-Cruzado, Vishal G. Shelat, Vladimir Khokha, Wagih Mommtaz Ghannam, Walter L. Biffl, Wietse Zuidema, Yasin Kara, Yoshiro Kobe, Zaza Demetrashvili, Ziad A. Memish, Zoilo Madrazo, Zsolt J. Balogh, Zulfu Bayhan

**Affiliations:** 1 Department of General, Visceral, Thoracic, Transplant and Pediatric Surgery, University Hospital of Giessen, Rudolf-Buchheim-Strasse 7, 35392 Giessen, Germany; 2 Department of Surgery, Macerata Hospital, Macerata, Italy; 3 Department of Anesthesiology, Heidelberg University Hospital, Heidelberg, Germany; 4 Department of Pulmonary and Critical Care Medicine, University Hospital of Giessen and Marburg Lung Center (UGMLC), University Hospital of Giessen, Giessen, Germany; 5 Department of General, Emergency and Trauma Surgery, Pisa University Hospital, Pisa, Italy; 6 Department of Emergency Surgery, Parma Maggiore Hospital, Parma, Italy

## Correction to: World Journal of Emergency Surgery (2022) 17:34 https://doi.org/10.1186/s13017-022-00424-0

Following the publication of the original article [1], the author name “Dragos Serban” under The WSES COVID-19 emergency surgery survey collaboration group was incorrectly written as “Dragos Seban” instead of “Dragos Serban”.

The original article has been corrected.
